# Targeting inorganic nanoparticles to tumors using biological membrane‐coated technology

**DOI:** 10.1002/mco2.192

**Published:** 2022-12-08

**Authors:** Yuanyuan Zhang, Qian Chen, Yefei Zhu, Manman Pei, Kairuo Wang, Xiao Qu, Yang Zhang, Jie Gao, Huanlong Qin

**Affiliations:** ^1^ Nanotechnology and Intestinal Microecology Research Center Shanghai Tenth People's Hospital, School of Medicine Tongji University Shanghai China; ^2^ Precision Medicine Center Taizhou Central Hospital Taizhou Zhejiang China; ^3^ Changhai Clinical Research Unit Shanghai Changhai Hospital Naval Medical University Shanghai China

**Keywords:** biological membrane, cancer therapy, immunomodulation, inorganic nanoparticles, targeted delivery

## Abstract

Inorganic nanoparticles have extensively revolutionized the effectiveness of cancer therapeutics due to their distinct physicochemical properties. However, the therapeutic efficiency of inorganic nanoparticles is greatly hampered by the complex tumor microenvironment, patient heterogeneity, and systemic nonspecific toxicity. The biomimetic technology based on biological membranes (cell‐ or bacteria‐derived membranes) is a promising strategy to confer unique characteristics to inorganic nanoparticles, such as superior biocompatibility, prolonged circulation time, immunogenicity, homologous tumor targeting, and flexible engineering approaches on the surface, resulting in the enhanced therapeutic efficacy of inorganic nanoparticles against cancer. Therefore, a greater push toward developing biomimetic‐based nanotechnology could increase the specificity and potency of inorganic nanoparticles for effective cancer treatment. In this review, we summarize the recent advances in biological membrane‐coated inorganic nanoparticles in cancer precise therapy and highlight the different types of engineered approaches, applications, mechanisms, and future perspectives. The surface engineering of biological membrane can greatly enhance their targeting, intelligence, and functionality, thereby realizing stronger tumor therapy effects. Further advances in materials science, biomedicine, and oncology can facilitate the clinical translation of biological membrane‐coated inorganic nanoparticles.

## Graphical abstract

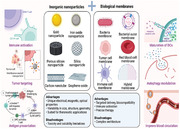

## 1 INTRODUCTION

Cancer is still the leading cause of mortality worldwide.^1^ Cancer treatment strategies include surgery, radiotherapy, and chemotherapy. However, drug resistance, limited surgical site, toxicity, and adverse side effects limit the effectiveness of cancer treatment, burdening society and the patients’ families.^2^ The use of synthetic material engineering in nanoparticles could improve diagnosis and drug delivery, resulting in enhanced antitumor activity.^3^ Inorganic nanoparticles have emerged as a promising tool in cancer treatment due to their unique physicochemical properties, such as acoustic, photo, and electrical properties. Compared with organic nanoparticles, inorganic nanoparticles possess unique advantages such as a large specific surface area, easy preparation, more uniform distribution, and distinct properties (such as acoustic and photoelectric properties).^4^ However, low biocompatibility and short circulation duration without modification are some major factors limiting the use of inorganic nanoparticles in clinical settings.^5^ Inorganic nanoparticles, such as gold, silver, and silica, have high load capacity and are used for drug delivery. However, their applications are limited, as they are nonbiodegradable.^6^ In addition, due to abnormal tumor metabolism, hypoxia, and low pH, inorganic nanoparticles are restricted in their use due to immunosuppression and drug resistance in the tumor microenvironment (TME). Therefore, altering the properties of inorganic nanoparticles to overcome their limitations facilitates the application of inorganic nanoparticles in cancer therapeutics.

Recently, the use of biomimetic nanoparticles, such as cell‐ or bacteria‐derived membranes (i.e., biological membranes), has shown tremendous potential as anticancer agents.^7^ These naturally derived biological membranes inherit their parent cell/bacteria's properties and functions, including tumor targeting, homologous cell adhesions, superior biodegradability, penetration of the cell membrane, and modification of cell surface molecules. Hence, these membranes can be used as safe and efficient in vivo vectors for targeted drug delivery.^8^ Coating inorganic nanoparticles with biological membranes confers advantages such as prolonged circulation, superior biocompatibility, tumor antigenicity, high target specificity, and minimum toxicity, making them a potential candidate for cancer therapy.^9^

Over the past decade, multiple studies have shown the significance of membrane‐coated nanoparticles.^10^ Biological membrane‐coated inorganic nanoparticles have attracted attention as promising candidates for cancer therapy. In the field of precise tumor therapy, researchers need to address three major challenges: improving biocompatibility, enhancing tumor targeting, and countering the immune escape mechanism of tumors with biological membrane‐coated inorganic nanoparticles. Engineering strategies can offer the following great opportunities for improving the efficacy of biological membrane‐coated inorganic nanoparticles.

**FIGURE 1:**
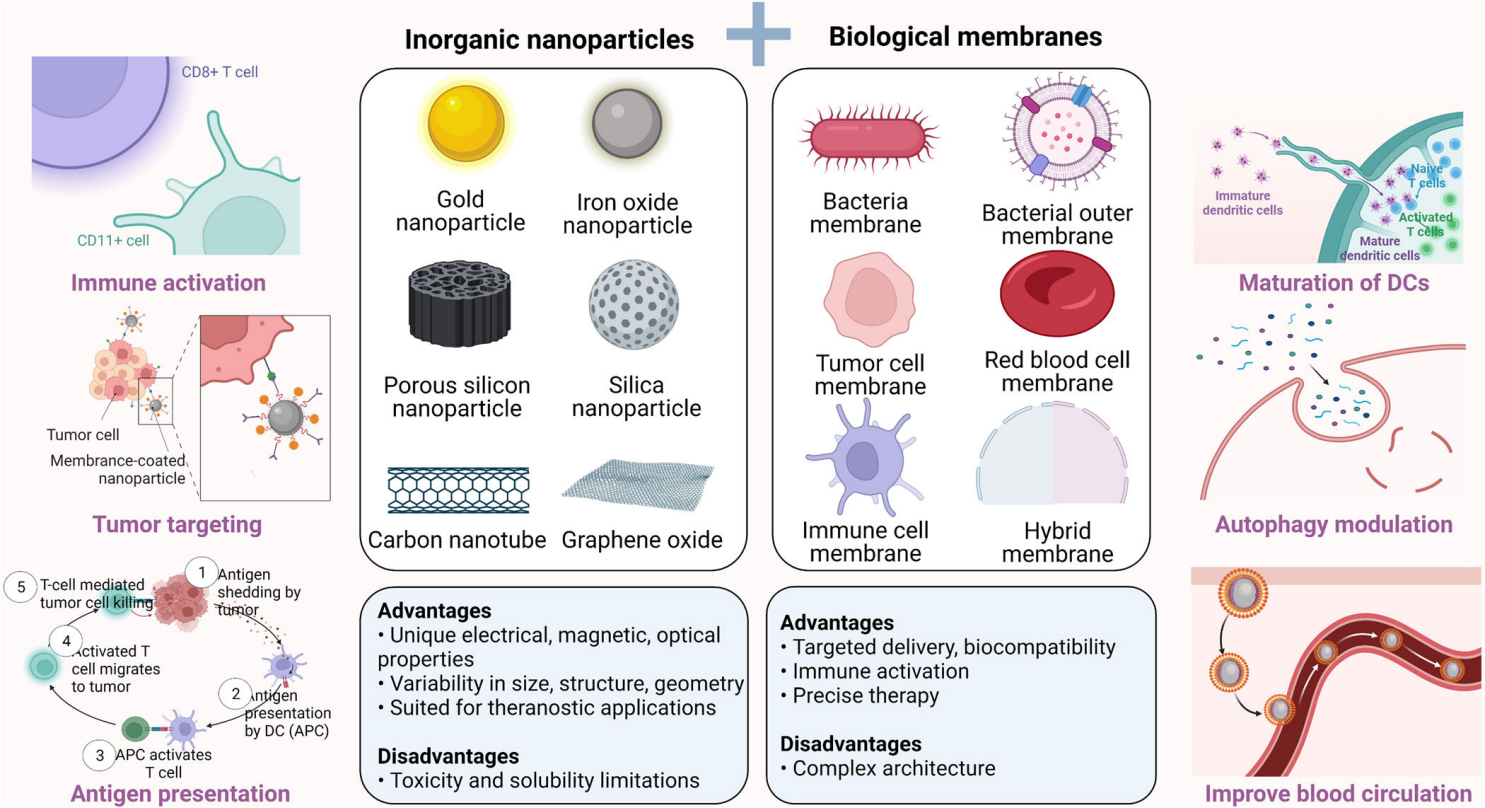
Schematic illustration of novel approaches for biomimetic precision antitumor strategies based on biological membrane‐coated inorganic nanoparticles. Created with Biorender.com

In this review, we summarized common engineering strategies and recent research progress based on the literature. We systematically summarized the design parameters and the applications of biological membrane‐coated inorganic nanoparticles in cancer therapy. As shown in Figure 1, biomimetic nanotechnology platforms combining biological membranes [such as tumor cell membranes, red blood cell (RBC) membranes, platelet membranes, immune cell membranes, mesenchymal stem cell (MSC) membranes, bacteria membranes/outer membrane vesicle (OMV), exosomes and hybrid membranes] and inorganic nanoparticles have been developed as potential candidates in cancer therapy. Finally, we discuss the advantages and disadvantages of different types of biological membrane‐coated inorganic nanoparticles for oncological treatment and clinical translation; current challenges in this regard have also been presented, revealing the state of progress and highlighting the potential for future research.

## 2 APPLICATION OF INORGANIC NANOPARTICLES IN TUMOR THERAPY

The combination of phototherapy and immunotherapy is considered to be an ideal treatment modality for primary and metastatic tumors. This synergistic treatment can not only improve the efficacy of the two therapies, but also overcome their inherent limitations, which opens a new era for anticancer treatment.^11^ In recent years, the development of nanoparticles has provided a platform for this treatment modality. Among all these nanoparticles, inorganic nanoparticles are ideal materials for this treatment modality because of their unique physicochemical properties.^12^ Inorganic nanoparticles are classified into metal, carbon, silicon, magnetic, and composite inorganic nanoparticles. They have been widely used in cancer treatment due to their physicochemical characteristics, such as optical, electrical, thermal, and magnetic properties, and exceptional performance in drug delivery.^13^

### 2.1 Metal nanoparticles

Metal nanoparticles (MNPs), including gold, silver, copper, iron, and other transition metals, have variable sizes and excellent acoustic and photoelectric properties. MNPs have attracted tremendous attention in cancer therapeutics due to their accuracy and are easy to control by altering their size, charge, and surface.^14^ Their unique acousto‐optic properties can be combined with photothermal therapy (PTT), photodynamic therapy (PDT), and sonodynamic therapy (SDT) for cancer treatment. Moreover, some MNPs have distinguished properties in antitumor treatment. For example, Ca^2+^ endocytosis in tumor cells causes mitochondrial dysfunction and induces immunogenic cell death.^15^ Mn^2+^ and Zn^2+^ can enhance the immune activation ability by activating the STING pathway to elicit potent innate and adaptive antitumor immunity for effective tumor suppression.^16^

### 2.2 Carbon‐based nanoparticles

Due to their photothermal properties, carbon‐based nanoparticles, such as graphene and carbon nanotubes (CNTs), have been widely used in cancer treatment. Graphene is a two‐dimensional nanomaterial related to carbon and has unique electrical, thermal, and mechanical properties, a large surface area, good biocompatibility and stability, and excellent electrical conductivity.^17^ CNTs are widely used in drug delivery due to their surface functionalization, easy control, and adjustable physical and chemical properties. For example, various antigens or adjuvants can be directly grafted onto the surface of CNTs or encapsulated in the internal structure by simple covalent modification. CNTs can be rapidly internalized by antigen‐presenting cells (APCs) and trigger effective immune responses.^18^

### 2.3 Silicon‐based inorganic nanoparticles

Silicon‐based nanoparticles are biodegradable and biocompatible. Hence, they are excellent platforms for various biomedical applications.^19^ Mesoporous silica nanoparticles (MSNs) are silica‐based nanomaterials with honeycomb‐ordered porous structures. MSNs are widely used in cancer treatment due to their excellent adsorption properties for various biological molecules,^20^ adjustable sizes and surface modifications, biocompatibility, and chemical and thermal stability.

### 2.4 Black phosphorus nanosheet

Among many nanoparticles, black phosphorus nanosheet (BPNS), as a new two‐dimensional layered inorganic nanoparticle, has been widely used in tumor therapy due to its excellent electrical conductivity, optical properties, and low toxicity.^21^ As a photothermal agent, it has been widely used in the photothermal treatment of preclinical tumors. BPNS can produce local hyperthermia under 808 nm laser irradiation, which can be used for PTT and drug release.^22^ It has shown good application prospects in photoacoustic imaging, PTT, PDT, drug loading, and other fields.

### 2.5 Layered double hydroxide

Layered double hydroxide (LDH), also known as natural hydrotalcite, is a layered crystalline nanocomposite material composed of divalent and trivalent metal cations.^23^ LDH is a new type of two‐dimensional inorganic nanomaterial that possesses a large specific surface area, significant surface electric charge, good biocompatibility, biodegradability, and hypotoxicity, so LDH has been used in a variety of biomedical applications, including cancer treatment, bioimaging, and antibacterial treatment.^24^

### 2.6 Composite inorganic nanoparticles

Composite inorganic nanoparticles have distinct properties and are usually synthesized by physical or chemical methods involving two or more inorganic materials with different physicochemical properties.^25^ Inorganic materials complement each other's performance in composite inorganic nanoparticles, resulting in the synergistic effect of all inorganic materials in multimodal therapy for cancer. Currently, there are some nanomaterials based on composite inorganic nanoparticles that have entered clinical trials, such as silver nanoparticle/calcium hydroxide nanoparticles, Cu/Zn nanoparticles, mixture of gold and silver nanoparticles, and zinc‐carbonate hydroxyapatite nanocrystals.^26^

## 3 DESIGN OF BIOLOGICAL MEMBRANE‐COATED INORGANIC NANOPARTICLES

### 3.1 Strategies for designing biological membrane‐coated inorganic nanoparticles

Biological membranes are composed of various functional proteins, phospholipids, and lipopolysaccharides. They have special structural and functional properties, which makes these membranes attractive biological candidates and can be used in combination with traditional synthetic materials.^27^ Thus, surface modification of inorganic nanoparticles using a variety of biological membranes has become a promising strategy to improve biocompatibility, extend the duration of circulation, and enhance the specificity of inorganic nanoparticles.^28^ They aid in cancer therapy by promoting immune recognition, enhanced immunogenicity, effector cell activation, and TME regulation.

### 3.2 Choice of biological membranes

Understanding the structure and function of biological membranes is essential in designing effective membrane‐coated nanoparticles. The cell membrane is composed of lipids, proteins, and carbohydrates and is semipermeable. The primary function of the cell membrane is to protect cell integrity through selective osmosis, where it selectively allows essential molecules to pass.^29^ The membranes form closed vesicles, which form a physical barrier between the inside and outside of the vesicles and can be used to design carriers for drug delivery and templates for nanoparticle synthesis. Moreover, some cells have an inherent ability to target abnormal tissues using recognition elements distributed on the cell membranes. The membranes that coat nanoparticles mainly include tumor membranes, immune cell membranes, RBC membranes, bacterial membranes (BMs), and hybrid membranes. Each membrane confers its unique functions to biomimetic nanoparticles, and their similarities and differences form the basis of their selection. For example, almost all biological membranes can improve nanoparticle biocompatibility. RBC membranes are used to increase the biocompatibility and circulation time of nanoparticles.^30^ Tumor and exosome membranes can enhance the specificity of drug delivery to tumor cells by nanoparticles,^31^ while immune cell membranes can stimulate the maturation of dendritic cells (DCs) and induce the anticancer immune response.^9^

### 3.3 Methods of membrane coating

Membrane coating is an ideal method to ensure the biocompatibility and performance of nanoparticles. It has aided in surpassing the limitation of traditional surface modification of nanomaterials in biomedical applications.^32^ The initial work depended entirely on physical extrusion, in which the nanoparticle core and the purified membrane are coextruded through a porous membrane.^33^ This approach is suitable for synthesizing liposomes, where the mechanical forces exerted through extrusion disrupt the membrane structure, allowing it to reassemble around the nanocore. Recently, a sonication‐based approach has also been adopted for membrane coating,^34^ where the two components are subjected to destructive force by ultrasonic energy, resulting in the spontaneous formation of core–shell nanostructures. Other novel membrane‐coated technologies utilized to encapsulate nanoparticles into cell membranes have also been reported.^35^ A microfluidic system combining rapid mixing and electroporation has been used to encapsulate magnetic nanoparticles with RBC membranes.^36^ Recently, microfluidic sonication has been reported to produce exosome membrane or tumor cell membrane‐coated nanoparticles encapsulated with imaging agents in a one‐step and straightforward manner.^37^, ^38^ By fine‐tuning the pulse voltage, duration, and flow rate of the microfluidic system, high‐quality nanoparticles with complete coatings and excellent stability were obtained.

## 4 APPLICATIONS OF BIOLOGICAL MEMBRANE‐COATED INORGANIC NANOPARTICLES IN CANCER THERAPY

To overcome these shortcomings and broaden the application of inorganic nanoparticles in the field of cancer treatment, the development of inorganic nanoparticles coated with biological membranes has been promoted. These materials not only retain the original characteristics of inorganic nanoparticles but are now endowed with the properties of biological membranes. Membrane‐coated inorganic nanoparticles are a novel modification that improves the biological safety of inorganic nanoparticles, prevents the aggregation of materials, and prolongs the circulation time of nanoparticles in blood.^39^ Some membranes use “the homing effect,” which increases the actual effective concentration of the drug and can be used to target the tumor site. The BM acts as an adjuvant, which wraps around the inorganic nanoparticles and fuses with the tumor cell membrane. This membrane‐coated nanoparticle can effectively target tumors and stimulate the immune system. In addition, inorganic nanoparticles can be endowed with more functions by coating the surface with hybrid membranes of different cell types (Table 1). Therefore, biological membrane‐coated inorganic nanoparticles are a promising new method in cancer therapeutics.^40^

**TABLE 1:** Summary of the differences among biological membranes

Biological membranes types	Targeting ability	Key features	Limitations	References
Tumor cell	Homologous targeting	Targeting homologous tumor cells stimulates tumor‐specific immunity	Short internal circulation	41, 42
Red blood cell	Reticuloendothelial system targeting	Prolong blood circulation, improve immune escape ability	May cause hemolysis	43, 44
Platelet	Targeting vascular wounds	Good immunocompatibility and efficient properties in homeostasis therapy	Complex synthetic and purification routes	45, 46
Immune cell membrane	Inflammatory site targeting	Immune escape and activation of dendritic cells maturation	Low blood levels in some tumors	47, 48
MSC	Tumor‐targeting capability	Low immunogenicity, tumor‐targeting ability and inflammatory migratory	The regulatory role played by MSC membrane in tumors is unclear	49, 50
Bacteria membrane/outer membrane vesicle	Adhesion targeting	Capture tumor neoantigen, stimulate antitumor T‐cell response	Immune storms caused by bacterial walls	51, 52
Exosome	Insert specific molecular targeting	The transporter secreted by cells to overcome biological barriers and evade immune surveillance	The protein integrity of the membrane is vulnerable	53, 54
Hybrid membrane	Multiple targeting	Achieve tumor personalized combination therapy	Complex preparation process	55, 56

Abbreviation: MSC, Mesenchymal stem cell.

### 4.1 Tumor cell membrane‐coated inorganic nanoparticles

Tumor cells can evade immune surveillance and target homologous cells due to specific proteins and receptors on their surfaces.^57^ Recently, an approach to cloaking tumor cells that takes advantage of homotypic adhesion and immune escape has drawn a great deal of interest.^58^ Tumor cell membrane‐coated inorganic nanoparticles can escape from macrophage uptake due to the presence of the membrane protein CD47, which helps tumor cells escape from the immune system, and adhere to tumor cells for self‐targeting and demonstrate strong intracellular uptake based on the adhesive properties of surface molecules.^59^ It can effectively induce antitumor immune responses in vivo.^41^ Therefore, coating the tumor cell membrane can extend the circulation time in the body, and greatly improve the homologous targeting ability of inorganic nanoparticles.^42^

**FIGURE 2:**
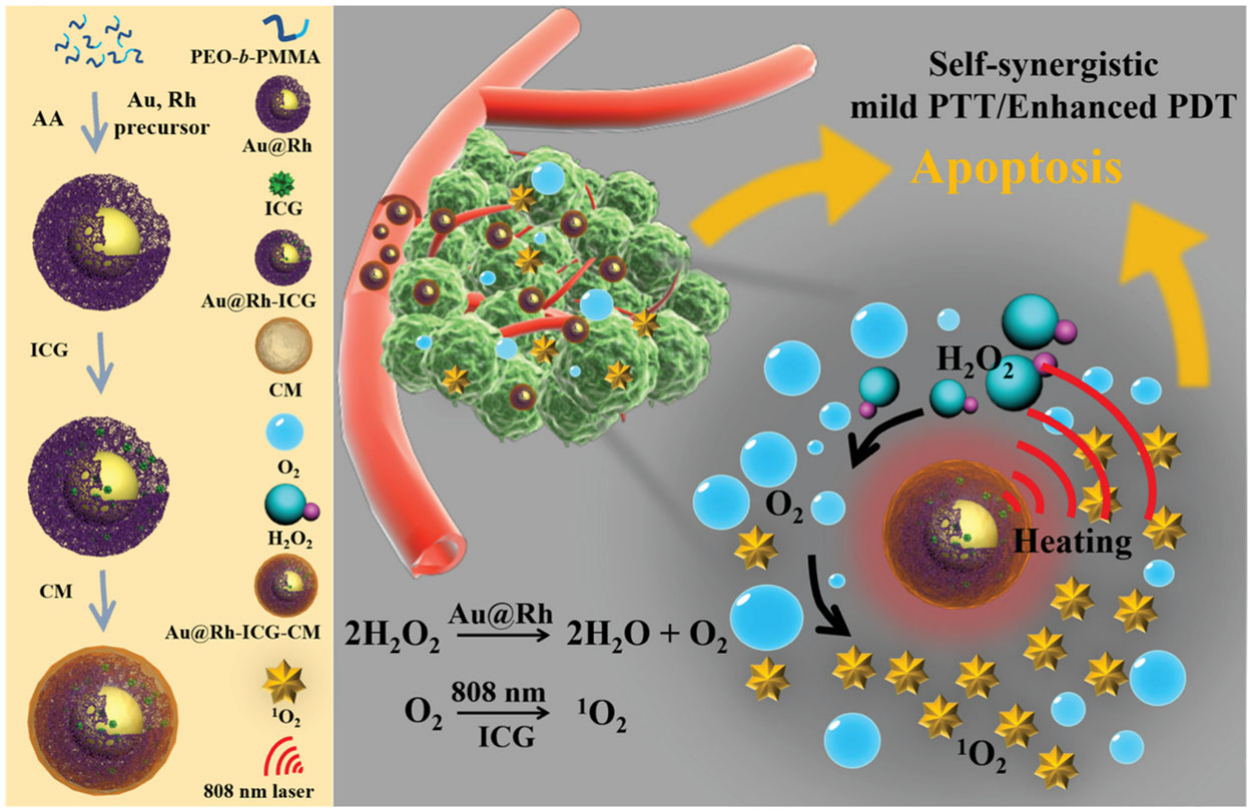
Schematic illustration shows the key steps in preparing porous Au@Rh core–shell nanostructures and their associated major mechanistic pathways in cancer therapy. Reproduced with permission from reference.^60^ Copyright © 2020, WILEY‐VCH Verlag GmbH & Co. KGaA, Weinheim

As shown in Figure 2, Wang et al. used tumor cell membranes to coat porous bimetallic nanostructures, which effectively reduced tumor hypoxia and achieved high PDT efficiency. This porous Au@Rh core–shell nanostructure exhibits catalase‐like activity, where it uses hydrogen peroxide from the tumors to catalyze oxygen production efficiently. Au@Rh nanostructures coated with tumor cell membranes can achieve tumor targeting through homologous binding.^60^ The specific antigens on the cancer cell surface help homologous cancer cells adhere to tumor tissue.^61^ Therefore, coating the surface of inorganic nanoparticles with the tumor cell membrane enriched the tumor tissue with the nanoparticles and achieved the expected drug delivery. Chao and coworkers used homologous targeting of the cancer cell membrane (CCM), immune escape mechanism, and the performance of iridium complexes to target mitochondria, constructed based on the black titanium dioxide cascade target multifunctional nanoplatform (Ir‐B‐TiO2@CCM), realized multimode imaging‐mediated photothermal and sonodynamic cotherapy, improved its antitumor efficiency, and provided a way and material for the efficient design of a nanoantitumor platform.^62^ Li and coworkers synthesized tumor membrane‐coated gold nanocages (AuNs) loaded with adriamycin. Nanoparticles were targeted to the tumor site to release drugs rapidly via photoheat, thereby killing the tumor cells via a thermal effect, and the combined chemotherapy and phototherapy effect was achieved. This is a promising drug delivery system that combines active targeting, stimulus‐responsive drug delivery, chemotherapy, and phototherapy.^63^ These examples confirm that inorganic nanoparticles coated with tumor cell membranes effectively induce homologous targeting in vivo. Moreover, Fang et al. reported a biomimetic nanovaccine coated with melanoma cell membranes to induce tumor‐specific immune responses. This nanovaccine, carrying the complete array of tumor cell membrane antigens, provides a powerful platform applicable to multiple anticancer therapeutic modalities.^64^

However, tumor antigens alone are not enough to counter the immunosuppressive effects of the TME. Furthermore, adding immunomodulatory molecules such as immunoadjuvants on the membrane surface can induce immune responses more efficiently, thereby conferring additional advantages to the nanoparticle. The antitumor efficacy of the nanoparticle was significantly enhanced by combining the PTT, PDT, and SDT of inorganic nanoparticles and immune therapy. Thus, coating inorganic nanoparticles with tumor cell membranes is widely accepted and can be applied to other tumor models. It could also be used as personalized medicine in cancer vaccination to prevent cancer recurrences, where the patient's tumor cell membrane can be the raw material.^65^

### 4.2 RBCs membrane‐coated inorganic nanoparticles

RBCs account for more than 99% of blood cells. Hemoglobin in RBCs binds with oxygen molecules and transports them from the lungs to different tissues in the body.^43^ Surface markers on RBCs protect them from being cleared by macrophages, thus prolonging their circulation time. Artificial antigen‐presenting cells (aAPCs) are used in cancer immunotherapy and function similarly to APCs, such as DCs, to activate T lymphocytes and generate antitumor responses. Furthermore, complement receptor (CR) proteins and immune‐related molecules on the RBC cell surface can act as aAPCs to regulate the immune microenvironment and induce antitumor immune responses.^44^ In conclusion, RBCs membranes can be easily degraded in vivo without generating toxic byproducts and are biocompatible, which makes them promising biological carriers for drug delivery.

**FIGURE 3:**
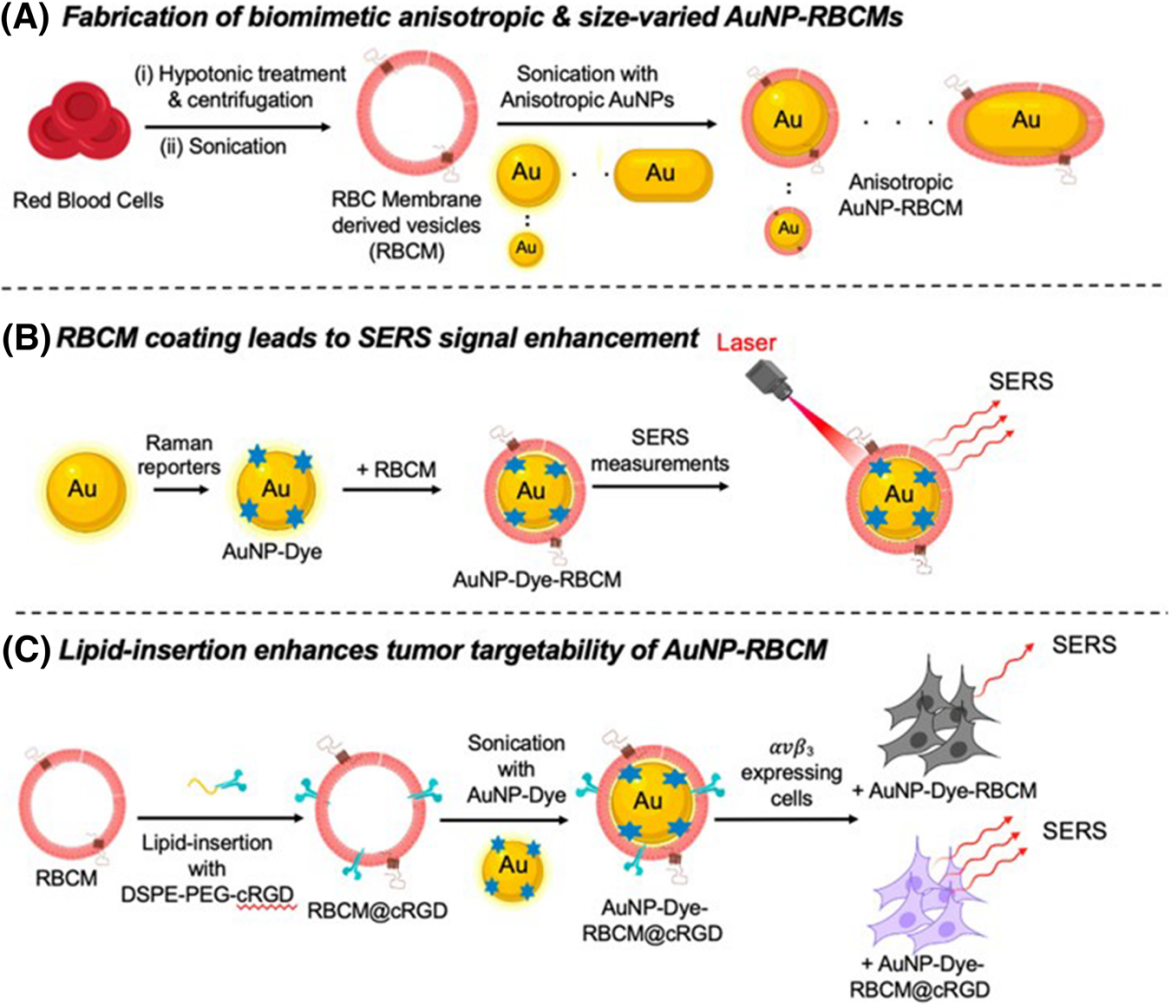
(A–C) Red blood cell membrane‐coated inorganic nanoparticles. Reproduced with permission from reference.^70^ Copyright © 2022, American Chemical Society

Piao et al. coated gold nanocages with RBC membranes, and the cells were killed using 850 nm infrared light by optothermal treatment. Interestingly, the blood circulation time of the coated nanocages significantly improved in vivo compared to the uncoated gold nanocages, showing a better therapeutic effect.^66^ RBC membrane‐coated nanoparticles showed prolonged circulation time, could better escape immune surveillance, and had enhanced tumor accumulation. Intravenously administered RBCs have a long circulating lifespan, which allows them to interact with T cells more effectively. Han et al. prepared an antigen delivery system based on RBCs (nano Ag@erythrosomes). The tumor antigen was loaded on the RBC membranes by fusion. The fusion of surgically removed tumor antigens and RBCs can be used to create personalized immunotherapy, which inhibits cancer recurrence and metastasis.^67^ Meanwhile, CD47 is a “do not eat me” marker on the surface of RBCs that evades immune clearance by interacting with receptors for signal‐regulating protein α.^68^ In addition to prolonging blood circulation and escaping immune clearance, RBC membranes can promote surface‐enhanced Raman scattering (SERS) effects. SERS nanoparticles with good biocompatibility and nontoxic SERS effects have attracted attention due to their applications in biomedical fields such as ultrasensitive in vitro diagnosis, in vivo tumor imaging, and spectroscopy‐guided tumor surgery.^69^ Nie and coworkers developed a simple and reliable method to improve the stability and dispersity of RBC membrane‐coated SERS nanoparticles (Figure 3). The signal intensity of SERS nanoparticles coated with RBC membranes was much higher than that of SERS nanoparticles coated with polyethylene glycol. This additional strength gained can be attributed to the hydrophobic nature of the hydrocarbon chains of the RBC membrane, which reduces electron damping and promotes electromagnetic field enhancement.^70^

In conclusion, RBC membrane‐coated inorganic nanoparticles showed prolonged circulation time, could escape immune clearance, gained antigen‐presenting ability and were more conducive to treating tumors, representing promising bionic nanoplatforms.

### 4.3 Platelets membrane‐coated inorganic nanoparticles

Platelets are small pieces of cytoplasm that have been cleaved from the cytoplasm of mature megakaryocytes in the bone marrow. Many studies have shown that platelets play a crucial role in the occurrence and development of malignant tumors. Recently, significant cross‐communication between platelets and tumor cells has been found.^45^ Tumor cells can be promoted to proliferate by platelets. Compared with RBC membranes, platelet membranes coatings have the advantage of targeting tumors and tumor sites. Surface ligands on platelet membranes, such as CD47, help nanoparticles escape clearance by the immune system. Moreover, multiple receptors on the surface of platelet membranes can also directly interact with specific components at the tumor site to achieve targeting effects. For instance, Rao et al. prepared a nanoparticle coated with platelet membranes to enhance the diagnostic and therapeutic effects of tumors. They collected platelet membranes from mouse blood and coated Fe_3_O_4_ magnetic nanoparticles with them. This nanoparticle elicits no immune response and has the ability to actively target tumors, magnetic resonance imaging and PTT.^71^ Nie and coworkers used MSNs coated with platelet membranes to combine vascular disrupting agents with antiangiogenic drugs to achieve targeted aggregation of both in tumors. Platelet membrane camouflage gives nanoparticles the ability to escape early systemic clearance and target tumors through receptor–ligand interactions between platelet membranes and tumor vasculature endothelial cells.^46^ Thus, in addition to increasing tumor site accumulation with the ability to target tumors, platelet membrane‐coated inorganic nanoparticles also reduce systemic toxicity. Platelet membranes have the potential to be one of the best candidates for bionic systems for cancer treatment due to their abundant source, low cost, simple extraction, and low immunogenicity.

### 4.4 Immune cell membrane‐coated inorganic nanoparticles

Studies have shown a relationship between immune cells, cancer progression, and treatment. Immune cells possess unique characteristics, such as tumor recognition and tropism induced by cell–cell interactions and tissue migration, especially the ability to cross physiological barriers, which enable immune cells to play their unique antitumor role.^47^, ^48^ The membranes derived from immune cells have functions similar to those of immune cells, such as tumor recognition. Thus, immune cell membrane‐coated inorganic nanoparticles have also been widely used in cancer therapeutics.

**FIGURE 4:**
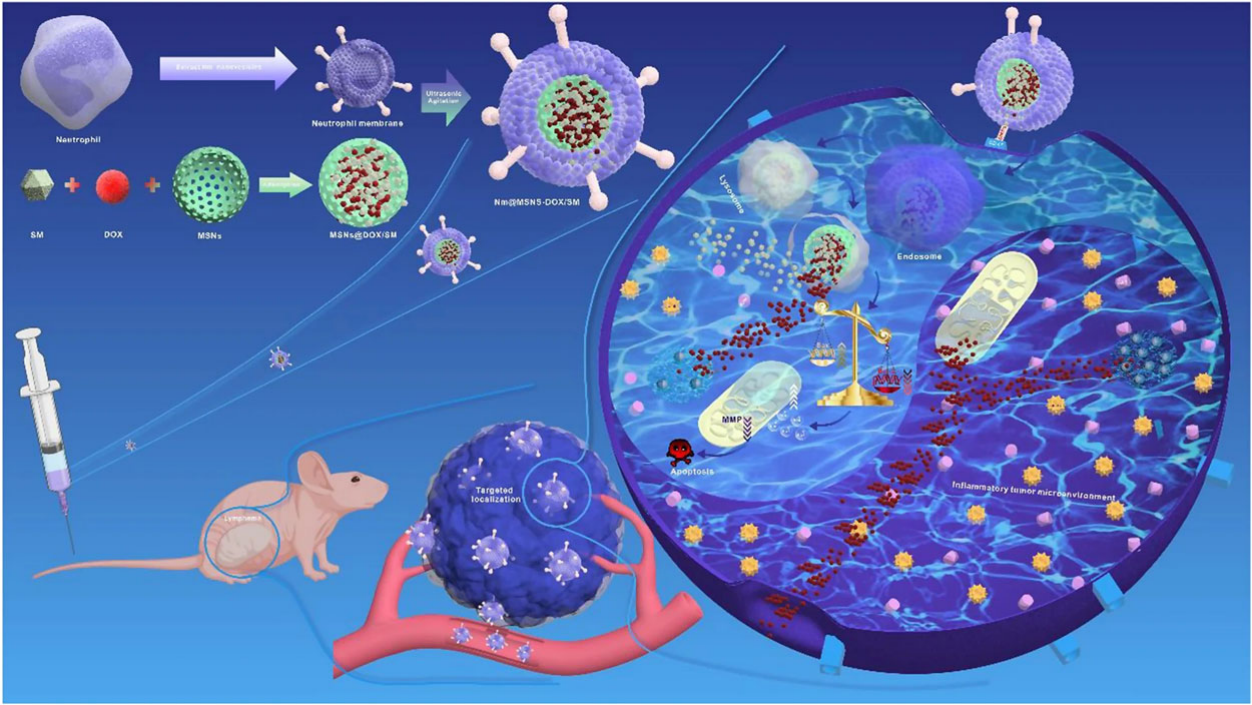
Schematic diagram of Nm@MSNs‐DOX/SM construction and its targeted therapeutic mechanisms in lymphoma. DOX, doxorubicin; MSN, mesoporous silica nanoparticles; Nm, neutral membranes; SM, Shanzhiside methylester. Reproduced with permission from reference.^73^ Copyright © The Author(s) 2021

Neutrophils are the first white blood cells recruited at the inflammatory site during infection and cancer. It accounts for 50%–70% of the human circulatory system and is the most abundant cell type in the TME.^72^ Therefore, neutrophil‐based nanoparticles may have various applications in diagnosing and treating inflammatory diseases and cancer. Neutrophils can be divided into two subtypes: N1 neutrophils have potent antitumor activity, and N2 neutrophils have tumor‐promoting functions. These inflammatory cells send out signals for the continuous recruitment of neutrophils. Inorganic nanoparticles coated with neutrophil membranes offer great opportunities for efficient drug delivery at cancer sites. Zhao et al. attempted to construct MSNs loaded with doxorubicin (DOX) and the anti‐inflammatory drug Shanzhiside methylester (SM). MSNs were used as the core of the nanoparticles, and neutral membranes (Nm) were used as the shells, resulting in the successful synthesis of Nm@MSNs‐DOX/SM (Figure 4).^73^ The results showed that Nm@MSNs‐DOX/SM evaded immune recognition, accumulated, and triggered drug release at the tumor site in a weakly acidic tumor environment, leading to the synergetic effect of DOX and SM in cancer therapeutics.

The macrophage membrane has a camouflage strategy that helps them achieve a superior half‐life in the systemic circulation and improve tumor adhesion ability without losing drug‐loading capacity or the nanosize advantage.^74^ The covering macrophage membrane promotes the circulation, stability, and tumor targeting of inorganic nanoparticles.^75^ By blocking the exposure of exogenous materials, macrophage membranes reduce nanoparticle toxicity and excretion by organisms to prolong circulation. Furthermore, membrane proteins provide tumor‐targeting effects.^76^ Moreover, the membrane coating of macrophages enables the inorganic nanoparticles to have the ability to cross the blood–brain barrier, which can be used for specific targeted therapy of orthotopic gliomas.^77^ Due to these advantages conferred by the cell membrane, various macrophage membrane‐coated nanoparticles have been developed in cancer therapy. Li and coworkers reported macrophage cell membrane (MPCM)‐coated gold nanoshells (AuNS) as a new generation of photothermal nanoconstructs for in vivo cancer PTT. Under the guidance of surface proteins on MPCM, prolonged circulation time and enhanced accumulation of MPCM‐AuNS were observed in vivo. When irradiated by near‐infrared light, tumor cells were ablated by thermal energy transferred by AuNSs.^78^ This biomimetic strategy significantly improved the efficacy of AuNS‐mediated PTT for cancer by integrating the advantages of near‐infrared radiation thermal effects produced by AuNSs with the prolonged circulation time and active recognition inherited from macrophages.

DCs are widely regarded as the most effective APCs. DCs acquire and process antigens, present them to T cells, and play an important role in activating the immune response. DCs have been widely used in developing vaccines and cancer immunotherapy.^79^ Studies have shown the effect of DC membrane‐coated inorganic nanoparticles. Zhang et al. developed immune gold nanoparticles (AuNPs), which were generated in cells and combined with PTT and immunotherapy through exocytosis. AuNPs were first created in melanoma B16F10 cells, followed by coating with DC membranes to form AuNPs (AuNP@DCB16F10). The biomimetic nanosystem could stimulate DC maturation and activate the T‐cell immune response, thereby effectively inhibiting primary, metastatic, and recurrent tumors and significantly improving the survival rate of tumor‐bearing mice.^80^

Each immune cell protects the body from foreign invaders (bacteria, fungi, and viruses) and diseases, including cancer. They can leave the circulatory system and penetrate deep into tissues, such as the inflammatory and hypoxic areas of tumors and inflamed brain tissue, which is impossible for inorganic nanoparticles alone to enter.^81^ APCs process and display surface antigens that help activate and recruit T cells that mediate the antitumor effect. These immune cells are activated and recruited to tumor sites in large numbers, thereby inducing immune reactions. Complex proteins on immune cell membranes play a major role in the recruitment of immune cells and mediating immune responses. Due to these distinct properties, inorganic nanoparticles coated with immune cell membranes can infiltrate the tumor, inhibit growth, and prevent tumor recurrence.

### 4.5 Mesenchymal stem cell membrane‐coated inorganic nanoparticles

MSCs are multifunctional stem cells that can differentiate into many cell types and can be isolated from a variety of tissues, including bone marrow, fat, umbilical cord, dental pulp, and placenta.^82^ MSCs also have strong cell proliferation and tissue repair capabilities, and are involved in the regulation of immune responses and tumor progression.^49^ MSCs have antitumor properties and can slow tumor growth by affecting immune cells.^83^, ^84^ However, some evidences suggest that MSCs can also promote tumor growth through immune regulation and TME remodeling.^85^ MSCs play a role in tumor progression by secreting relevant factors and hormones that act on target tissues.^50^ The unique properties of MSCs are mainly due to their membranes containing a variety of receptors, including cytokine receptors, chemokine receptors, growth factor receptors, cell matrix receptors, and receptors for cell–cell interactions. Therefore, MSC membranes are an ideal potential vehicle for establishing drug delivery systems.

**FIGURE 5:**
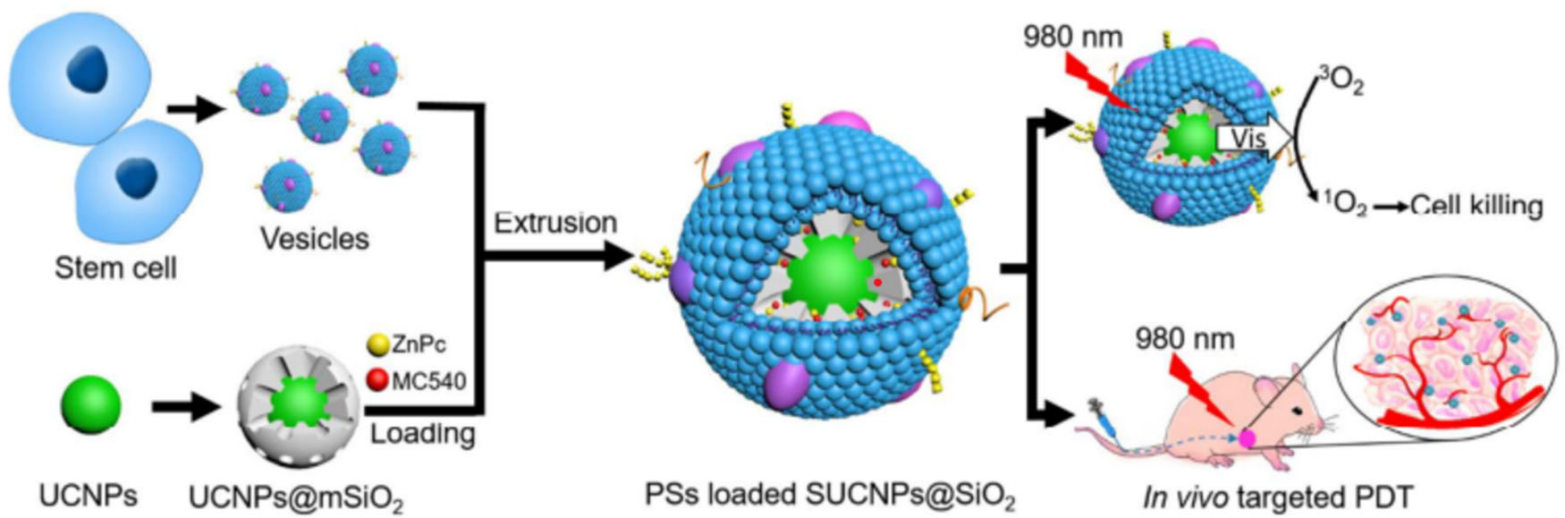
Fabrication process of UCNPs@mSiO2 and their mechanism in photodynamic therapy (PDT). Reproduced with permission from reference.^87^ Copyright © 2016, American Chemical Society

Gao and coworkers reported MSC membrane‐coated MSN (MSN@M) that possessed the capability of active stealth and self‐positioning drug delivery, and solved the related problems of MSCs‐mediated drug delivery. MSN@M showed stronger tumor targeting and penetration than MSN in tumor‐bearing mice. At the same time, MSN@M showed strong MSN drug‐loading ability and continuous drug release ability when loaded with DOX.^86^ The upconversion nanoparticles (UCNPs) based PDT agents are promising for deep‐tissue cancer treatment. He and coworkers developed MSC membrane‐coated MSN loaded with photoinitiators (UCNPs@mSiO2) as a PDT nanoplatform (Figure 5). Taking advantage of the advantages of MSC membranes, such as tumor targeting and long retention capacity, this novel nanoplatform exhibited significant accumulation at the tumor site.^87^ In conclusion, MSC membrane‐coated nanoparticles are susceptible to being targeted and homing by ligands overexpressed on the MSC membrane. MSC membrane‐coated nanoparticles provide a wide range of options for developing targeted drug delivery, showing the potential to enhance therapeutic efficacy in the treatment of various diseases.

### 4.6 Bacterial membrane‐coated inorganic nanoparticles

Bacteria are single‐celled microorganisms with unique structural, physiological, and evolutionary characteristics. The antitumor action of bacteria is fundamentally based on their unique biological properties, which makes them very suitable as anticancer agents or vectors.^51^ The directed movement of bacteria in response to environmental stimuli is called bacterial tropisms, such as chemotaxis, phototaxis, thermotaxis, pH tropism, and oxygen tropism.^52^ These properties confer the bacteria with natural tumor‐targeting capabilities, allowing them to migrate from distant sites to the tumor site, selectively colonize tumors, and even infiltrate inaccessible tumor regions with an oxygen gradient.^88^ While living bacteria have the potential to be used as microrobots for targeted delivery and production of biological agents, they are accompanied by safety concerns such as potential immunogenicity and pathogenicity.^8^ Therefore, bacterial structures such as membranes and OMVs are often used in cancer therapy. This BM is composed of polysaccharides, lipids, proteins, and other virulence factors that can be used for antitumor research. Multiple studies have shown the application of BM‐coated organic nanoparticles in cancer treatment.^89^ However, only a few studies have reported the role of BM‐coated inorganic nanoparticles in cancer therapy. Morris and coworkers prepared a multifunctional bacterial membrane‐coated nanoparticle (BNP). BNP consists of four components: an inner polymeric nucleus composed of PC7A, CpG oligonucleotides, and an outer layer composed of the *Mycobacterium smegmatis* membrane modified with maleimide groups to enhance antigen uptake. After radiotherapy, *M. smegmatis* membranes can capture tumor neoantigens and promote antigen uptake and processing by DCs, simulating the antitumor T‐cell response, which inhibits tumor growth and generates tumor‐specific memory cells.^90^ Therefore, it is tempting to predict that further research on BNPs will make a breakthrough in cancer therapeutics.

**FIGURE 6:**
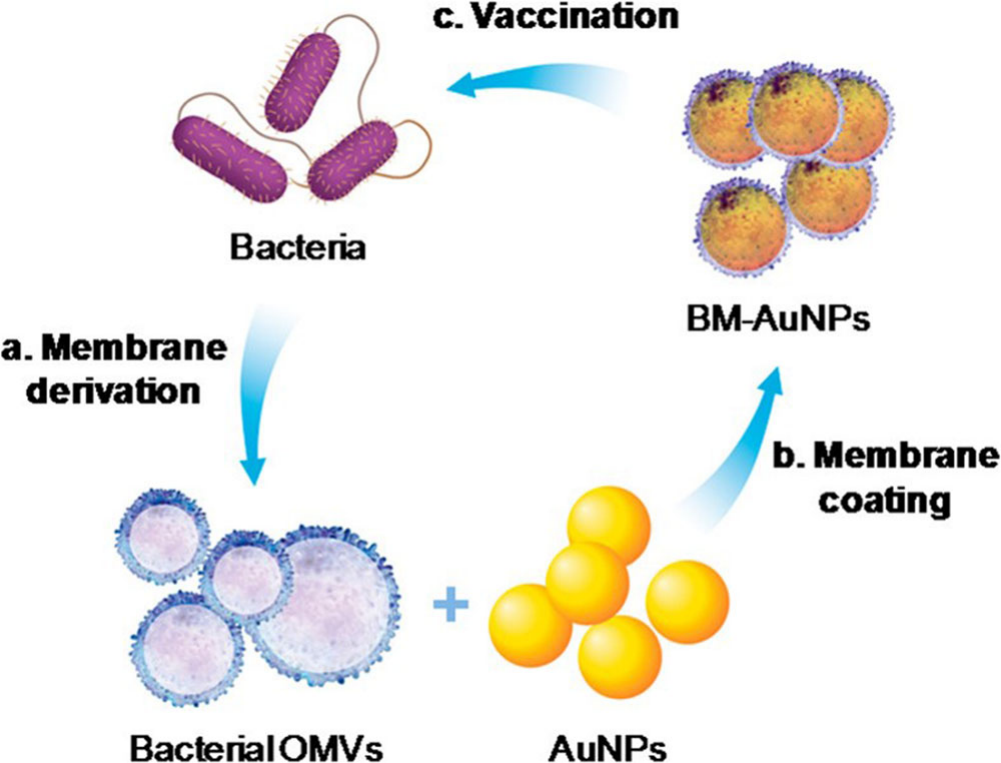
A schematic illustration of the modulation of antibacterial immunity via bacterial membrane‐coated nanoparticles. Reproduced with permission from reference.^94^ Copyright © 2015, American Chemical Society

In addition to the BM, OMVs also have unique advantages in enhancing drug stability, targeting, and biocompatibility.^91^ OMVs are extracellular carriers ubiquitously expressed around gram‐negative bacteria and have strong natural bactericidal activity.^92^ OMV retains key features of BMs and components of DNA, RNA, proteins, metabolites, and toxins, thus preserving endocytosis‐mediated targeting and internalization capabilities. Efforts have been made to develop OMVs as a good platform for delivering antibacterial drugs. Studies have shown the use of OMVs in developing vaccines or immune adjuvants.^93^ Gao et al. reported a BM‐coated nanoparticle system known as a novel antibacterial vaccine (Figure 6). This nanoparticle was created by collecting bacterial OMVs from *Escherichia coli* as a model pathogen. This OMV was used to coat AuNPs with a diameter of 30 nm, which improved the stability of BM‐AuNPs. Additionally, the rapid activation and induction of DC maturation could provide a new strategy for the synthesis of antibacterial vaccines using OMVs.^94^

The above examples show that BNPs can target tumor tissues or specific locations based on their performance characteristics, thereby overcoming the limitations of inorganic nanoparticles. BMs or OMVs encapsulate specific components such as polysaccharides, lipids, proteins, DNA, RNA, and pilus. These can act as neoantigens and promote DC uptake, thereby stimulating antitumor T‐cell reactions, which significantly inhibit tumor growth and generate antitumor immunity. Therefore, inorganic nanoparticles coated with BMs or OMVs act as “artificial bacteria” in cancer therapy. Combining them with immunotherapy and inorganic nanoparticle‐based therapy can produce a more efficient and controlled treatment system.

### 4.7 Exosome‐coated inorganic nanoparticles

**FIGURE 7:**
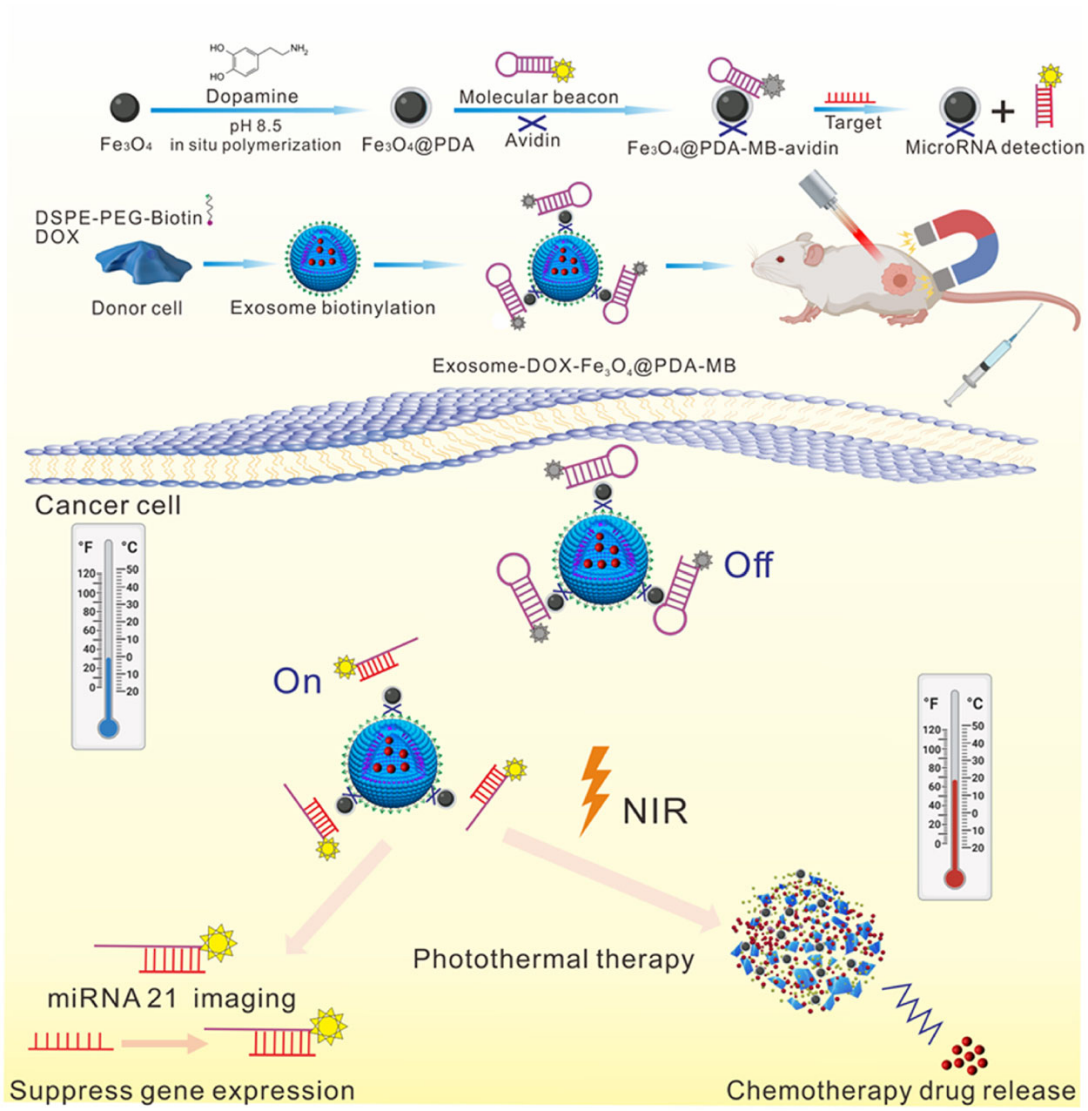
Schematic illustration of the design of Exo‐DOX‐Fe3O4@PDA‐MB as a robust nanoplatform for targeted delivery and chemo/gene/photothermal tumor therapy under near‐infrared (NIR) irradiation. DOX, doxorubicin. PDA, polydopamine. MB, molecular beacon. Reproduced with permission from reference.^97^ Copyright © 2021, Elsevier Ltd. All rights reserved

Exosomes are small extracellular vesicles secreted by cells and are used as nanoparticles due to their stable nature, biocompatibility, low immunogenicity, and toxicity.^53^ In addition, exosomes are effectively taken up by cells and have homing ability, which depends on their membrane proteins.^54^ Typically, exosome‐biomimetic nanoparticles are constructed by iterative physical extrusion or freezing/thawing cycles to fuse exosomes and nanoparticles. This may affect protein integrity on exosome membranes, thereby impairing the biological function of these biomimetic nanoparticles.^95^ Hence, there is a need to develop an effective method for generating exosome‐bionic nanoparticles without altering the membrane integrity for cancer therapy. Yong et al. constructed porous silicon nanoparticles (PSiNPs) coated with exosomes (secreted by tumor cells), which can be used for targeted drug delivery and to kill cancer stem cells. When tumor cells are incubated with DOX‐loaded nanoparticles (DOX@PSiNPs), they secrete exosomes (DOX@E‐PSiNPs) containing DOX@PSiNPs. In vivo results showed that DOX@E‐PSiNPs can aggregate in large quantities at the tumor site and exhibit anticancer activity in subcutaneous, in situ, and metastatic tumor models. This result indicates that biomimetic exosome‐based nanoparticles can be used as an efficient drug delivery vector for tumor chemotherapy.^96^ Liu and coworkers devised a strategy to combine biocompatible exosomes with chemical/genetic/photothermal therapy (Figure 7). The platform consisted of exosomes loaded with DOX coated on magnetic nanoparticles and combined with molecular beacons that target miR‐21 for responsive molecular imaging. Exosome‐coated magnetic nanoparticles were guided by the external magnetic field, which allowed the accumulation of exosomes at the tumor site. Local hyperthermia was induced by near‐infrared radiation, which subsequently triggered the release of the exosome payload. This exosome‐mediated multimodal therapy is a promising and efficient platform for precision cancer nanomedicine.^97^

In conclusion, exosomes are endogenous nanosized particles secreted by various cells and absorbed by recipient cells because of their unique structure and composition. They are less cytotoxic, overcome biological barriers, can escape immune surveillance and have been used as multifunctional therapeutic vectors for the diagnosis and treatment of cancer. Furthermore, exosomes have high stability in the blood during circulation due to their stable lipid bilayer membrane and similar cellular composition. Due to this, they can stabilize encapsulated nucleic acids and proteins and improve hydrophobic drug solubility. Moreover, the overexpression of CD47 protein on the exosome membrane helps them escape phagocytosis by macrophages. Furthermore, some studies have found that PD‐L1 on exosomes derived from tumors can help tumor cells escape immune surveillance.^98^ Exosome‐coated inorganic nanoparticles combined with multiple therapeutic methods could revolutionize cancer treatment. This review presents a new approach for creating theranostic nanoplatforms for precision medicine based on exosomes.

### 4.8 Hybrid membrane‐coated inorganic nanoparticles

**FIGURE 8:**
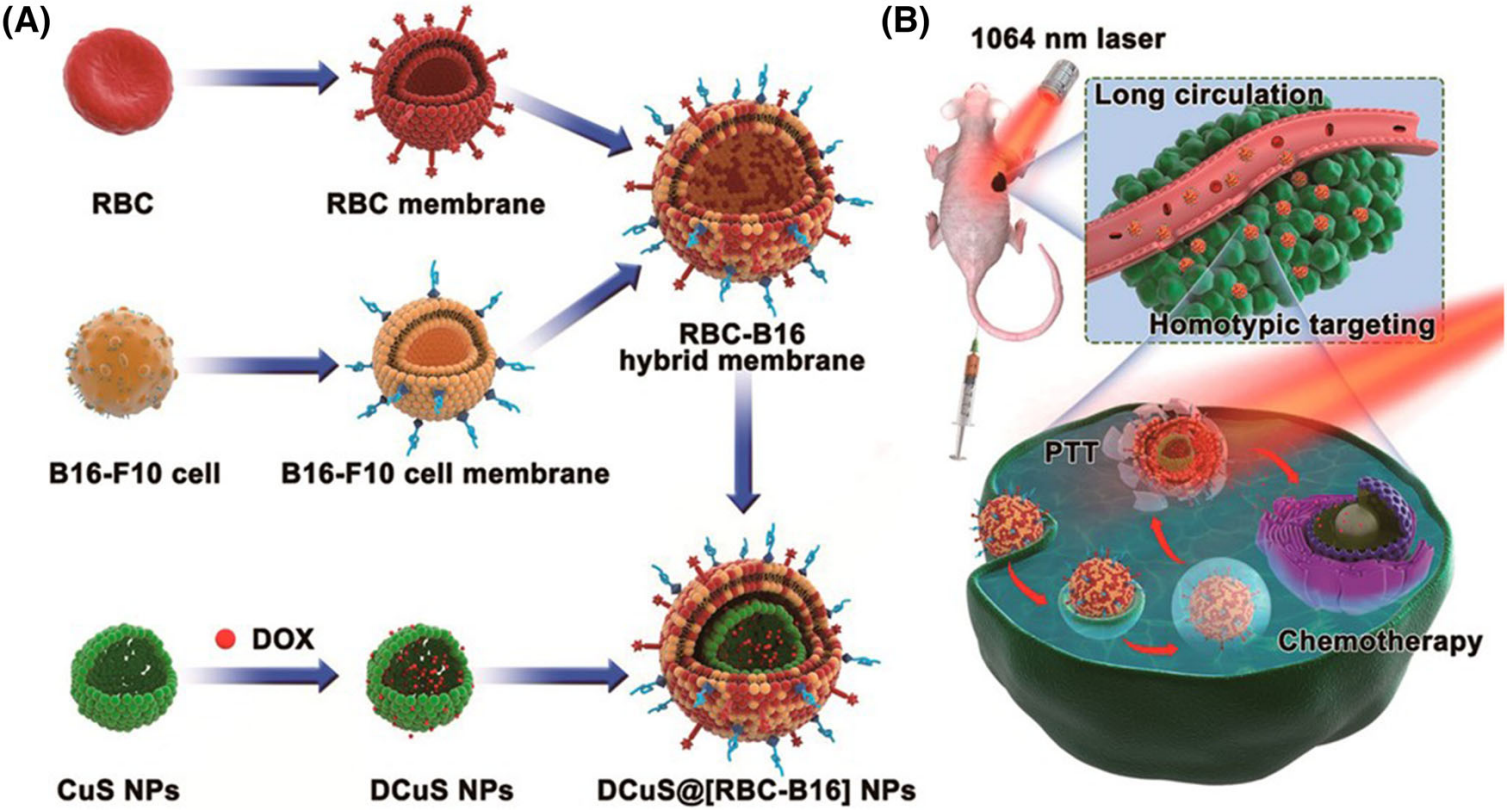
(A and B) Schematic of membrane fusion and coating for synergistic photothermal/chemotherapy for melanoma. Reproduced with permission from reference.^55^ Copyright © 2018, American Chemical Society

As mentioned above, inorganic nanoparticles coated with RBC immune cells and bacterial and tumor membranes can target tumor cells. The fluidity of the cell membrane makes it easy to combine different types of cell membranes and enhance tumor targeting. Zhang and coworkers fused RBCs and melanoma cell membranes (B16‐F10) to create a hybrid bionic coating (BC‐B16). Hollow copper sulfide nanoparticles (DCuS@[RBC‐B16] NPs) coated with RBCs and B16‐F10, mixed membrane camouflaged with DOX, were prepared (Figure 8). The bionic nanoparticles could preferentially recognize the source cells, had a prolonged life span during circulation and could specifically target homologous tumor cells in vivo. Thus, the combination of membranes and nanoparticles could effectively treat melanomas.^55^ Jiang et al. coated melanin nanoparticles with hybrid RBCs and breast cancer cell membranes. This allowed the homologous targeting of cancer cells by nanoparticles and improved photothermal efficacy. The results reveal that the fused hybrid membrane vesicles retained both RBC and MCF‐7 membrane proteins, and the composite nanoparticles had extended blood circulation time and homologous targeting of cancer cells. The homologous targeting function of nanoparticles was significantly enhanced by adding MCF‐7 membrane components to the hybrid membrane. However, increasing RBC membrane components to a hybrid membrane could effectively reduce the uptake of composite nanoparticles by macrophages and prolong their blood circulation time.^56^

Hybrid membrane nanoparticle vaccines are composed of tumor cell membranes displaying tumor antigens and BMs that have adjuvants on the surface of nanoparticles. The BM component of the vaccine elicits an external “danger signal” to the immune system, enabling immune cells to recognize tumor cell membranes, thereby improving the efficiency of tumor antigen presentation and activating the immune system. Nie and coworkers combined *E. coli* and autologous tumor cell membranes and coated nanoparticles to develop new personalized cancer vaccines. The results showed that these hybrid membrane nanoparticles induced a strong tumor‐specific immune response after surgical resection, which improved the mouse survival rate and prevented tumor recurrence.^99^ Tumor heterogeneity often leads to metastasis and alters treatment efficacy. Therefore, personalized treatment aims to target the tumor heterogeneity issue. In light of this information, Zhang and coworkers designed a vesicular system to enhance the innate immune response and designed personalized immunotherapy using bacterial OMVs with the tumor‐derived cell membrane (mT) to form new functional vesicles (mTOMVs). The results reveal that mTOMV enhances the activation of innate immune cells, specifically cleaves T cells in homologous tumors, and can accumulate in inguinal lymph nodes, thus inhibiting lung metastasis.^100^ However, personalized vaccines created by inorganic nanoparticles coated with hybrid membranes of tumor cell membranes and BMs have yet to be studied. This hybrid can trigger a specific string antitumor immune response, effectively inhibiting tumor recurrence and prolonging postoperative survival. Moreover, the superior performance of vaccines made of membrane‐inorganic nanoparticles provides a new perspective on personalized combination therapy.

The recurrence and metastasis of tumors after resection are closely related to the immune status. Specific activation of antitumor reactions without any significant side effects and enhanced immunogenicity by adjuvants can be a promising approach to overcome this limitation. Currently, personalized therapy has been proposed to combat tumor metastasis and recurrence issues by designing a hybrid membrane system. The hybrid system uses various membrane components to coat the nanoparticles, enhancing the innate immune response and strengthening the personalized immunotherapy response. This method can induce a strong and specific antitumor immune response, which effectively inhibits tumor recurrence, induces tumor regression, and prolongs postoperative survival. Moreover, the excellent photothermal properties and loading capacity of inorganic nanoparticles can be combined with various therapeutic methods. Therefore, this hybrid membrane‐coated inorganic nanoplatform has enhanced treatment potential in complex TME settings. Examples of biological membrane‐coated inorganic nanoparticles are summarized in Table 2.

**TABLE 2:** Summary of biological membrane‐coated inorganic nanoparticles for cancer therapy

Membrane types	Cell/bacteria types	Design	Preparation strategy	Key advances	References
Tumor cell	Human breast cancer cells	Breast cancer cell membrane‐coated Au@Rh nanostructures	Sonication	Tumor targeting through homologous binding	60
	Human cervical cancer (HeLa) cells	Tumor cell membrane‐coated black TiO_2_	Extrusion	Multimodal imaging‐mediated photothermal and acoustic dynamic cotherapy was realized and its antitumor efficiency was improved	62
	4T1 mouse breast cancer cells	Tumor cell membrane‐coated AuNs loaded with doxorubicin	Extrusion	The drug delivery system combines active targeting, stimulus‐responsive drug release, chemotherapy and phototherapy	63
RBC	RBC	RBC membrane‐coated gold nanocages	Extrusion	Prolong blood circulation, improve immune escape ability, significantly enhance the cumulative effect in the tumor site	66
		RBC membrane‐coated Ag nanoparticles	Extrusion	Antigen delivery system for personalized immunotherapy	67
		RBC membrane‐coated SERS nanoparticles	Sonication	RBC membranes give it a longer retention time and enhance its ability to escape immunity	70
Platelet	Platelet	Platelet membrane‐coated Fe_3_O_4_ magnetic nanoparticles	Extrusion	Elicit no immune response and has the ability to actively target tumors	71
		Platelet membrane‐coated MSNs nanoparticles	Sonication	Escape early systemic clearance and target tumors through receptor–ligand interactions	46
Immune cell	Neutrophil	Neutrophil membrane‐coated mesoporous silica nanoparticles	Extrusion	Evade immune recognition and accumulate actively at the tumor site	73
	Macrophage	Macrophage membrane‐coated gold nanoshells	Extrusion	Prolong the circulation time in vivo and enhance the accumulation in tumors, using PTT to treat tumors	78
	DC	DC membrane‐coated gold nanoparticles	Co‐incubation	Effectively activate T cells, activate cellular immunity, inhibit primary/metastatic/recurrence tumor, significantly improve survival rate	80
MSC	MSC	MSC membrane‐coated MSN	Sonication	Active stealth and self‐positioning drug delivery	86
		MSC membrane‐coated MSN	Extrusion	Tumor targeting and long retention capacity	87
Bacteria	MS	MS membrane‐coated BNP	Extrusion	Capture tumor neoantigen and promote DC uptake, stimulating antitumor T‐cell response	90
	*Escherichia coli* OMV	*E. coli* OMV‐coated gold nanoparticles	Extrusion	Improve stability in vivo and can activate and induce DC maturation rapidly	94
Exosome	Tumor‐derived exosome	Exosome‐based PSiNPs	Co‐incubation	Targeting tumor cells and tumor stem cells, penetrating deep into the tumor	96
	Macrophage‐derived exosome	Exosome‐based Fe_3_O_4_ NPs	Co‐incubation	Combined chemo/gene/photothermal therapy with tumor‐targeted exosomes as vectors	97
Hybrid membrane	RBC and melanoma cell	Hybrid membrane‐coated CuS nanoparticles	Sonication	Priority to identify source cells, prolong circulation time, and achieve specific targeting of homologous tumor cells in vivo	55
	RBC and breast cancer cell	Hybrid membrane‐coated melanin nanoparticles	Extrusion	Achieve extended blood circulation time and homologous targeting of cancer cells	56
	Bacterial and tumor cell	The integration of *E. coli* cell plasma membrane and autologous tumor cell membrane into nanoparticles	Sonication/extrusion	Stimulate specific antitumor immune response, effectively inhibit tumor recurrence, prolong the postoperative survival period	99
	Bacterial and tumor cell	Bacterial OMV hybridized with tumor cell membrane to form new functional vesicles	Sonication/extrusion	Enhance activation of innate immune cells and specific lysis of T cells in homologous tumors	100

Abbreviations: AuNs, gold nanocages; BNP, bacterial membrane‐coated nanoparticle; DC, dendritic cells; MS, *Mycobacterium smegmatis*; MSC, mesenchymal stem cell; MSN, mesoporous silica nanoparticle; NPs, nanoparticles; OMV, outer membrane vesicles; PSiNPs, porous silicon nanoparticles; PTT, photothermal therapy; RBC, red blood cell; SERS, surface‐enhanced Raman scattering.

## 5 MECHANISM UNDERLYING THE CELL MEMBRANE ENHANCING THE ANTITUMOR EFFECT OF NANOPARTICLES

### 5.1 Extended blood circulation time

Normal healthy cells are protected from immune attack because the biological membrane contains an “identity card.” Biological membranes act as camouflage to protect nanomaterials from immune system attack, allowing nanomaterials coated with cell membranes to circulate for extended periods.^101^ Nanomaterials with a long circulation life span have a significant clinical impact by systemic delivery of drugs to a specific target. In addition to lipids and proteins, RBC membranes contain natural stabilizers of nanomaterials.^102^ For example, RBCs can move freely between blood vessels and tissues without being attacked by the immune system due to the presence of CD47 on the membrane surface, which is recognized as self by the immune system.

### 5.2 Improving targeting capability

Nanoparticles have an excellent therapeutic effect; however, their adverse side effects have drawn significant attention. The targeting ability of nanomaterials is dependent on their affinity for target cells. Biological cell membranes have recognition elements that differentiate them from foreign or abnormal cells. Nanomaterials coated with these biological membranes inherit the ability of the membranes, which prevents them from being attacked by the immune system. Nanoparticles coated with macrophage membranes improved drug delivery to the tumor by binding to tumor cells via interactions between macrophage membranes and vasculature. Various leukocytes, such as natural killer (NK) cells and neutrophil membranes, have also been used to deliver precise treatment to tumors.^9^ NK‐cell membrane‐coated nanoparticles can induce proinflammatory M1 macrophage polarization to enhance antitumor immunity and specific tumor recognition. Nanoparticles coated with tumor cell membranes can target homologous tumors, referred to as homologous targeting. The active tumor‐targeting ability was preserved when the genetic material was replaced with membrane proteins.

### 5.3 Improving the immunogenicity of nanoparticles

Numerous antigenic motifs in biological membranes can be used to create vaccines without endangering genetic materials. Some immunogenic antigens found on BMs have intrinsic adjuvant capabilities and pathogen‐associated molecular patterns that can elicit both innate and adaptive immune responses. Combining the advantages of BMs and synthetic nanoparticles, BNPs could induce strong antibacterial immune responses. The tumor cell membranes contain many antigenic motifs, which can also be used to develop vaccines and improve immunity without causing cancer. However, the tumor cell membrane expresses only a portion of tumor antigens; hence, it can induce limited antitumor immune responses. Biological membrane‐coated nanoparticles are used to enhance the effectiveness of therapeutics by antigen delivery. Recently, biomimetic nanoparticles derived from biological membranes have been developed for antigen delivery for cancer immunotherapy. These membranes coating these nanoparticles are derived from RBCs, tumor cells, immune cells, bacteria, and exosomes. These biological membranes play a significant role in the antitumor effect. The tumor antigens displayed on the cell membrane are recognized and attacked by the immune system. Biomimetic nanoparticle formulations have been developed to efficiently present antigens to APCs, especially DCs, and induce an antigen‐specific T‐cell response.

## 6 CONCLUSIONS AND FUTURE OUTLOOK

Innovative bioengineering and nanotechnology strategies have led to the development of advanced biological materials for high‐performance tumor therapy. In this review, we have focused on recent advancements in the development of biological membrane‐coated inorganic nanoparticles, such as tumor membranes, RBC membranes, immune membranes, BMs, hybrid membranes, and exosomes. The exploration of biological membrane‐coated inorganic nanoparticles could be a new trend in designing nanoparticles for cancer therapeutics and can help enhance the outcomes of various cancers in the future. The emergence of novel biomimetic nanoplatforms has improved the delivery of inorganic nanoparticles to the tumor site. Biomimetic membrane coatings from different source cells/bacteria present a facile way of introducing multiple components onto the same inorganic nanoparticle without requiring complicated synthetic techniques.

Biological membrane‐coated inorganic nanoparticles have made remarkable progress in cancer treatment. However, several concerns need to be addressed before their implementation in therapeutics. Up to now, there have been no clinical trials of biological membrane‐coated inorganic nanoparticles for cancer treatment. Therefore, improving the biocompatibility and safety of biological materials is an important issue. Furthermore, due to tumor heterogeneity, the TME is strongly immunosuppressive and drug‐resistant, which can cause several off‐target effects and block the antitumor effects of biological substances. To enhance the performance of biomimetic nanoparticles, it is possible to incorporate a comprehensive biological component that will have synergistic behavior and possess synergistic features. For example, using antibodies, peptides, and proteins as ligands in cancer immunotherapy can enhance functionality. In addition, large‐scale production of biological membrane‐coated inorganic nanoparticles with consistency and reproducibility remains a challenge in a clinical setting. Source materials, such as RBCs, platelets, and leukocytes are readily available through well‐established protocols and infrastructure. The most practical approach will probably be to obtain and bank material from type‐matched donors rather than autologous sources. Techniques for large‐scale culture will need to be adapted for other cell types. Many such technical problems hinder the development and practical application of biological membrane encapsulation. For example, before membrane coating, inorganic nanoparticles tend to aggregate, which makes subsequent coating more difficult. After coating, the separation and purification of the product remain a problem that requires urgent attention.

Advances in bioengineering and nanotechnology have led to the development of new and advanced biological materials for cancer therapeutics. The rapid emergence of bioengineered therapeutic agents with anticancer properties, such as motility, tumor chemotaxis, tumor homing or colonization, immunomodulatory capacity, and cytotoxic potential, can significantly improve targeted drug delivery and antitumor immunity. The combination of nanotechnology and bioengineering has led to the generation of novel biological nanomaterials for efficient and precise cancer treatment. Natural biological membranes combined with inorganic nanoparticles have advantages exceeding their individual components. They complement the shortcomings of each component and bridge the gap between biological components and nanomaterials. This will help expand the range of traditional biomedical materials in cancer treatment and can be used to overcome tumor‐induced immunosuppression. These new engineered strategies will provide new possibilities for customized drug delivery and precision medicine.

Inorganic nanoparticles coated with biological membranes are one of the most promising candidates in personalized medicine. The technology mainly uses the special function of the biological membrane and integrates synthetic nanocarriers and bionic nanocarriers for disease treatment. At present, nanoparticles coated with biological membranes have been widely used in tumor treatment, toxin removal, and antibacterial applications and have achieved excellent results compared to traditional drugs. This is mainly due to the excellent biocompatibility of inorganic nanoparticles endowed by biological membrane coatings. The special biological functions of various membranes can be combined with synthetic nanomaterials to personalize treatment. Biological membrane‐coated inorganic nanoparticles decrease the gap between synthetic nanomaterials and biological systems to further inspire cancer immunotherapy approaches from a biological perspective. As this emerging biomimetic nanotechnology develops further, it will be a serious challenge to translate such platforms to the clinical setting for effective treatment and improving human health.

## AUTHOR CONTRIBUTIONS

The manuscript was mainly designed by H.L.Q., Y.Z., and J.G. Y.Y.Z. wrote the manuscript. M.M.P., K.R.W., and X.Q. contributed to the investigation. Q.C. and Y.F.Z. provided revisions to the content of the manuscript. All authors reviewed the manuscript, contributed to its content, and approved the final manuscript.

## CONFLICT OF INTEREST

The authors declare they have no conflicts of interest.

## ETHICS STATEMENT

Not applicable.

## Data Availability

Not applicable.
